# Revising of the Purcell effect in periodic metal-dielectric structures: the role of absorption

**DOI:** 10.1038/s41598-019-46071-5

**Published:** 2019-07-03

**Authors:** Konstantin M. Morozov, Konstantin A. Ivanov, Daniel de Sa Pereira, Christopher Menelaou, Andrew P. Monkman, Galia Pozina, Mikhail A. Kaliteevski

**Affiliations:** 1 ITMO University, 197101 St. Petersburg, Russia; 2 St. Petersburg Academic University, 194021 St. Petersburg, Russia; 3 Physics Department, Durham University, Durham, DH1 3LE UK; 4 Department of Physics, Chemistry and Biology (IFM), Linköping University, SE-58183 Linköping, Sweden; 5 Ioffe Institute, 194021 St. Petersburg, Russia

**Keywords:** Nanophotonics and plasmonics, Photonic devices, Nanophotonics and plasmonics

## Abstract

Periodic metal-dielectric structures attract substantial interest since it was previously proposed that the spontaneous emission amplification rates (the Purcell factor) in such structures can reach enormous values up to 10^5^. However, the role of absorption in real metals has not been thoroughly considered. We provide a theoretical analysis showing that absorption leads to diminishing values of Purcell factor. We also suggest that using emitting organic compounds such as CBP (4,4-Bis(N-carbazolyl)-1,1-biphenyl) can lead to a moderate increase of about an order of magnitude in the Purcell factor. Defining the experimentally measured Purcell factor as a ratio between the excited state lifetimes in bare CBP and in periodic structure, this increase in the fabricated periodic structure is demonstrated through a 4–8 times decrease in excited state radiative lifetime compared to a bare organic material in a wide emission spectrum.

## Introduction

Recently, metallic nanostructures supporting plasmonic resonances have been attracting significant interest due to unique properties caused by a strong light-matter interaction^1–4^. Surface plasmon (SP), which is a localized state of an electromagnetic filed at the metal-dielectric interface, has been theoretically predicted more than half a century ago^5^. Localization of the electromagnetic field at metal-dielectric interfaces allows the implementation of various sub-wavelength optical devices^6,7^, while the enhancement of the field’s magnitude near the interface can be utilized for different sensing techniques^8–11^ and in novel applications based on plasmonic effects^12–16^. The modification of the light-matter coupling can result in enhancement (Purcell effect)^17^ or in reduction of spontaneous emission rates which can improve the properties of light emitting of photovoltaic devices^18–21^.

Recently, the giant value of the Purcell factor was suggested in periodic metal-dielectric structures^22,23^. Also, it was proposed that a high spontaneous emission rate in such structures can be achieved through the high density of states (DOS), similar to the case of nanostructures with a high refractive index^24^.

However, latter conclusions have been doubted^25^ because in metals, absorption will hardly allow to utilize the benefits of the peculiar mode dispersion in the frequency range where the divergence in DOS happens for an ideal non-absorbing structure. Huge value of DOS associated with SP resonance is accompanied by absorption in plasma SP, which is not the case for all dielectric nanostructures^24^. Furthermore, previous results^22^ were obtained using formalisms, which works only within the light cone for dielectric layers in metal-dielectric structures. However, the major contribution to DOS is provided by the regions of the phase space outside the light cone. At the same time, the idea to use peculiar mode dispersion in the periodic metal-dielectric structures^26^ seems to be very compelling. Therefore, it is important and timely to provide a detailed analysis of the spontaneous emission rate in metal-dielectric structures taking into account absorption in metals and compare theoretical results with experimental studies.

Thus, in this work we study the influence of the absorption in metals on the modification of spontaneous emission in periodic metal-dielectric structures. To compare our model with the pre-existing model, where absorption in metals is neglected, we consider the structure identical to that analyzed by Iorsh *et al*.^22^.

## Results and Discussion

**Figure 1:**
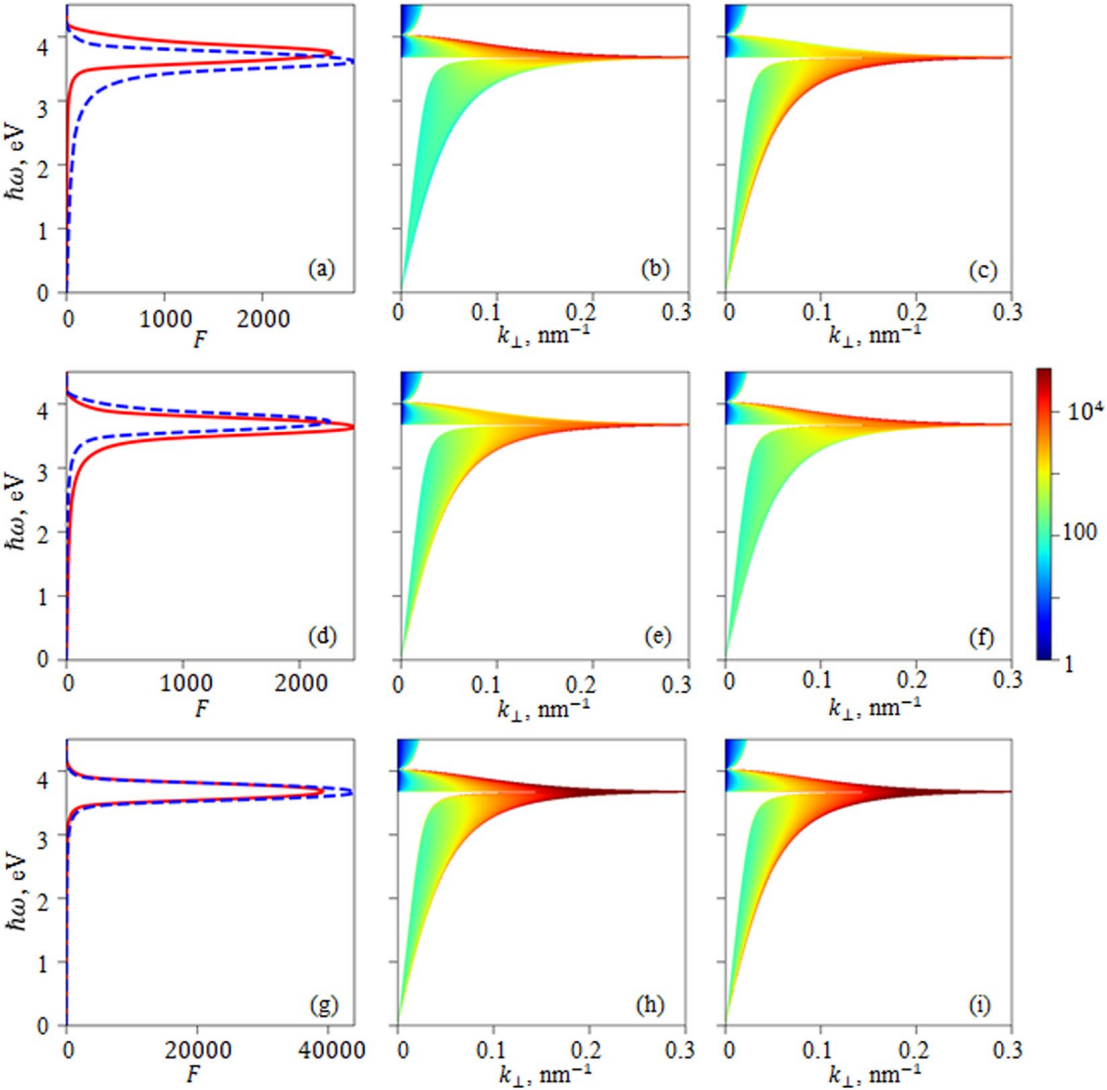
Integral Purcell factor (**a,d,g**) spontaneous emission probability density shown by color pattern (**b,c,e,f,h,i**) calculated for an infinite periodic structure of 15 nm Drude-modelled silver with no absorption/15 nm vacuum. The position of the emitting dipole is as follows: middle of the vacuum layer (top row); middle of the silver layer (middle row); layers interface (bottom row). Dipole orientation is as follows: parallel to the layers (red curve and middle column); perpendicular to the layers (blue curve and right column).

First, we have calculated the probability density of spontaneous emission for infinite idealized structures, for which we have assumed absence of absorption in silver. Such approximation is necessary for investigating of electromagnetic modes structure of infinite periodic structure. The period of the structure consisted of two layers: 15 nm of silver and 15 nm of vacuum. We limit our consideration to the case of TM polarization because SP and its associated features in the dispersion relations responsible for the increase of DOS and spontaneous emission rate can be realized only for TM polarization of light. The dispersion relations *hω*(*K*) and the spatial profile of the electromagnetic mode can be calculated using Bloch theorem (see chapter calculation technique of the section “Methods”). The resulting band structure is shown in Fig. 1. Plasmonic bands, positioned outside the light cone near the frequencies of SP of single silver-vacuum interface are clearly seen. SP and associated plasmonic band are waveguided modes, therefore, formally, there are solution for any wavevector *k*_⊥_. At the same time, since concentration of electrons in the plasma is finite, we should confine the interval of wave vectors, which are considered by the value corresponding to the mean inverse distance between electrons in the plasma, which is about one third of inverse nanometer for silver. Based on the dispersion relation and the spatial profile of electromagnetic modes, one can calculate the probability density of spontaneous emission (Equation 10) for specific wavevector, and then, integrating it over *k*_⊥_one can get the Purcell factor. Figure 1b,c,e,f,h,i show the dependence of the probability density on the wavevector *k*_⊥_ by color pattern. It can bee seen, that major contributions to the Purcell factor is provided by the states with high values of *k*_⊥_. Integrating the probability density of spontaneous emission, and normalizing it to the probability of the emission in the free space, one can obtain the Purcell factor, demonstrating pronounced peaks near the frequencies of SP on single interface.

To analyze the effect of absorption, we compare here results obtained for silver, which were used in our experimental studies and in previous theoretical work^22^.

The dielectric constant in metals according to the Drude theory can be written as follow:

1 $${\varepsilon }_{2}={\varepsilon }_{b}-\frac{{\omega }_{p}^{2}}{\omega (\omega -i\gamma )}$$

Where for silver *ε*_*b*_ = 4.96, $\hslash {\omega }_{p}=8.98$ eV and $\hslash \gamma =0.018$ eV^27^. However, the Drude theory fails within the frequency range which is particularly important for plasmon phenomena.

**Figure 2:**
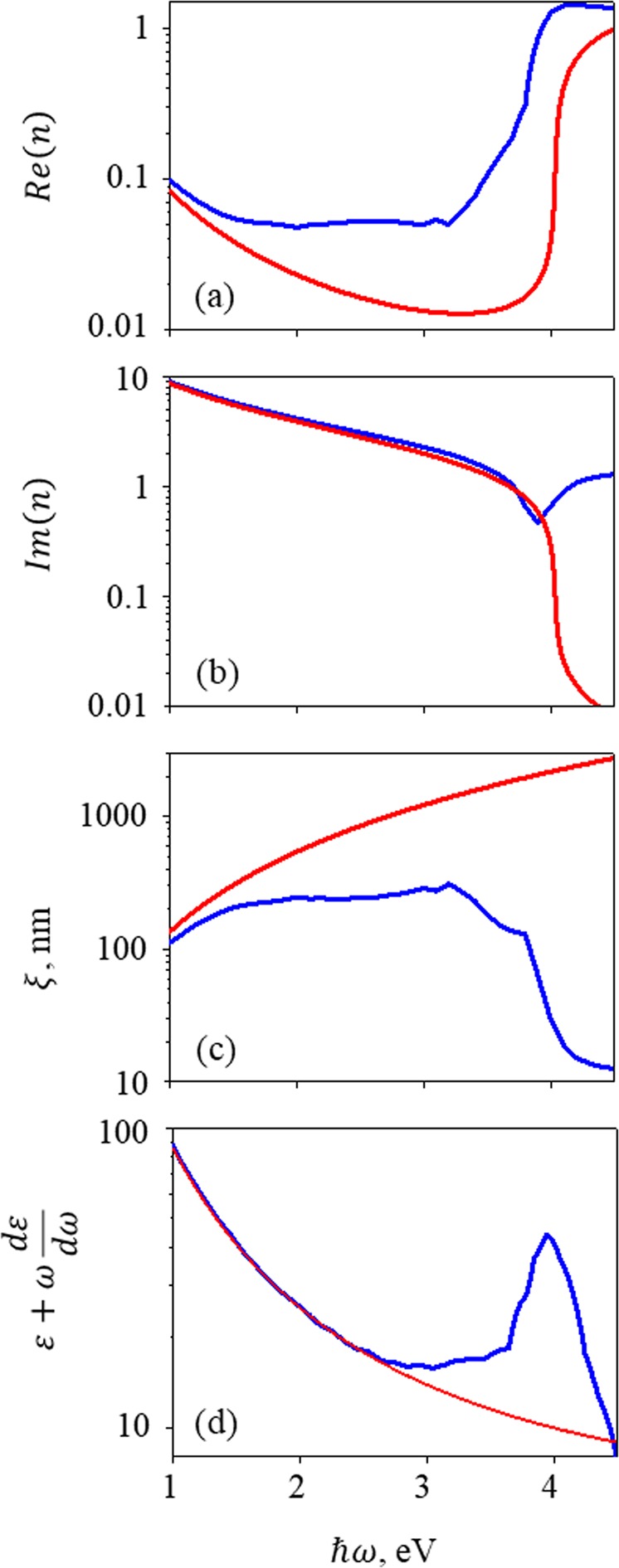
Real (**a**) and imaginary (**b**) parts of dielectric constant, coherence length (**c**) and energy density (**d**) for silver modelled according to the Drude theory with *ε*_*b*_ = 4.96, $\hslash {\omega }_{p}=8.98$ eV and $\hslash \gamma =0.018$ eV (red) and measured in experiment (blue).

Figure 2 illustrates the comparison of the dielectric constant approximated by the Drude model with the dielectric constant measured experimentally for real silver^28^. It is clear that the Drude model is inappropriate for the higher frequency range. The main difference is not related to the refractive index but to the definition of the coherence length:

2 $$\xi =\partial A/\partial z$$

Here *A* is the absorption coefficient. The coherence length for real silver is between 10 and 100 nm in the energy range of 3–4 eV. These values are much smaller than predicted by the Drude model, which leads to a significant reduction of any collective effects on scales beyond that length. Moreover, the energy density in metals is proportional to *ε* + *ωdε*/*dω*, whose value (plotted in Fig. 2d) differs dramatically for real silver.

The difference between the Drude model and experimental values becomes even more drastic for the plasmon dispersion curve. Indeed, from the dispersion equation we get the plasmon wavevector:

3 $${k}_{\perp }=\frac{\omega }{c}\sqrt{\frac{{\varepsilon }_{1}{\varepsilon }_{2}}{{\varepsilon }_{1}+{\varepsilon }_{2}}}$$

**Figure 3:**
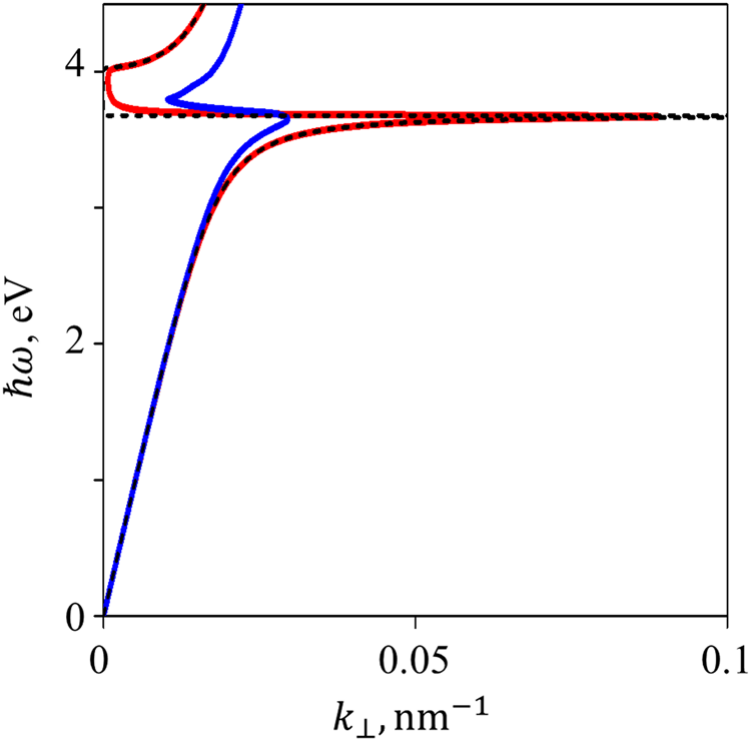
Plasmon dispersion for an interface between vacuum and silver modelled after the Drude theory without absorption consideration (black); with absorption consideration (red); for experimentally studied silver (blue).

Here *ε*_1_ and *ε*_2_ are dielectric constants of materials forming the interface. Comparison between a dispersion curve calculated using the Drude model and for real silver is shown in Fig. 3. We point out here that ignoring absorption in the Drude model leads to a situation when states with any wavevector *k*_⊥_ are available; at the same time, there is a forbidden zone for energy:

$${\omega }_{p}/\sqrt{{\varepsilon }_{1}+{\varepsilon }_{b}} < \omega  < {\omega }_{p}/\sqrt{{\varepsilon }_{b}}.$$

The situation changes completely when absorption is taken into account. Now, the spectrum is continuous in the whole frequency range, but there is a limit on the values of the wavevector *k*_⊥_. Numerically, its value doesn’t exceed 0.1 nm^−1^ and 0.05 nm^−1^ for the Drude-modelled and experimentally measured silver, respectively.

**Figure 4:**
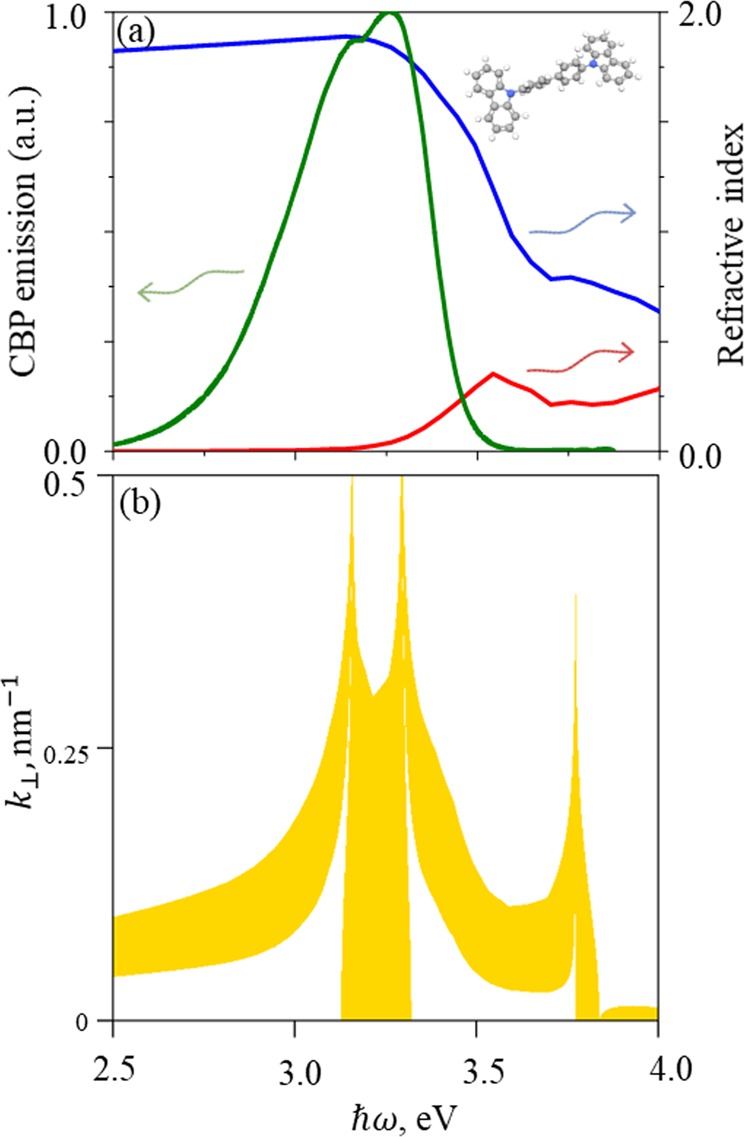
(**a**) Real (blue) and imaginary (red) parts of CBP’s refractive index; PL spectrum of CBP (green). Inset shows molecular structure of CBP. (**b**) Plasmonic band structure for an infinite structure of 15 nm CBP/15 nm silver (band allowed for surface plasmon marked yellow).

For experimental structure, it is important to choose a suitable material that will serve as an emitter instead of vacuum layers. Currently, organic emitting materials are considered as very promising in the field of photonics^29^. CBP (4,4-Bis(N-carbazolyl)-1,1-biphenyl) is one of the most popular host materials for different types of organic light emitting diode (OLED) systems including thermally activated delayed fluorescence devices due to its relatively large band-gap and ambipolarity^30,31^. We have chosen CBP as the dielectric emitting organic material due to the fact that the interface between CBP and silver allows SP within the CBP emission region (see Fig. 4a for the CBP refractive index, PL spectrum and molecular structure scheme; the calculated plasmonic band structure is shown in Fig. 4b). Co-occurrence of the spectral position of the SP on the silver/CBP interface and the emission band of CBP could be used for development of efficient organic light-emitting diodes for the ultraviolet region.

It should be noted that attempts to use organic compounds as light emitters in periodic structures have been  reported previously, however, without success in achieving high values of the Purcell factor^32^. The possible reason for this result can be related to the fact that the emitting material was only deposited on the surface of the structure. Contrary, we use an emitting organic material as the dielectric layer in each period of the structure.

**Figure 5:**
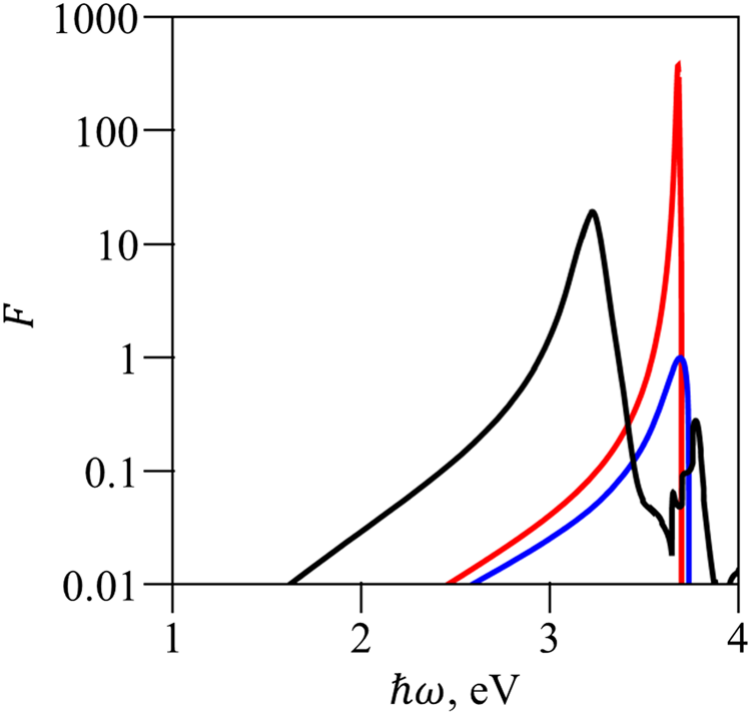
Purcell factor calculated for the interfaces between various materials: the Drude-modelled silver/vacuum (red); experimentally observed silver/vacuum (blue); experimentally observed silver/CBP (black). The emitter is placed at the interface and oriented parallel to it.

Moreover, theoretical calculations of the Purcell factor for a single metal-dielectric interface have been performed for three different configurations: vacuum/silver (according to the Drude model), vacuum/silver (after experimental work)^28^ and, finally, for CBP/silver (after experimental work)^28^. The results are presented in Fig. 5. It can be seen that calculations with real silver accounts for a dramatic decrease in the Purcell factor (by more than two orders of magnitude), while using CBP has several effects such as a shift of the peak amplification away from a small coherence length region, an increase of the Purcell factor value to about 10 and broadening the spectrum.

**Figure 6:**
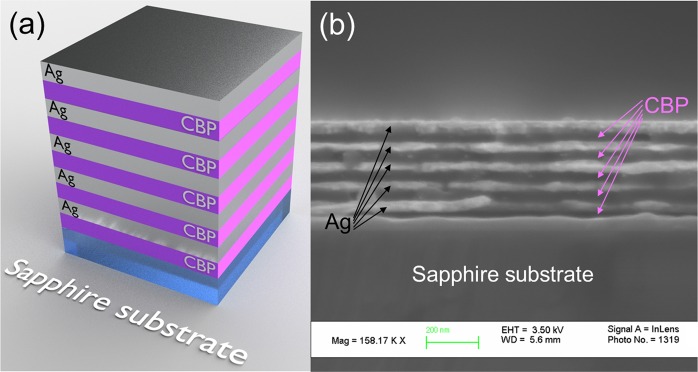
(**a**) Schematic drawing of the periodic CBP/Ag structure. (**b**) Cross-section SEM image of the 30 nm CBP/15 nm Ag structure at the sapphire substrate.

In order to approve our theoretical analysis, we have fabricated a series of test structures with the different CBP layer thicknesses (10 nm, 15 nm, 20 nm and 30 nm) and with a silver layer with a thickness of 15 nm deposited on Al_2_O_3_ (sapphire) substrate (see Fig. 6a illustrating an example of the test structure). Cross-section scanning electronic microscopy (SEM) image of the 5 period 30 nm CBP/15 nm Ag is shown in Fig. 6b. To get SEM images, the sample was cracked, which affected interfaces; however, it is clear that the interfaces between CBP and silver achieved by this fabrication method are rather smooth.

**Figure 7:**
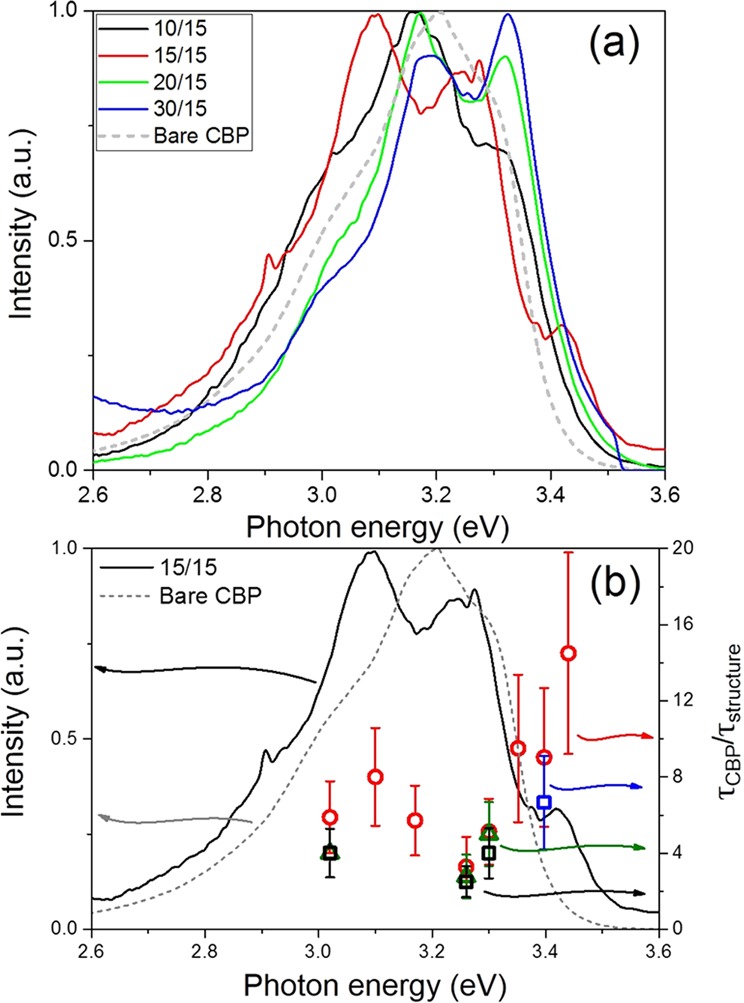
(**a**) PL spectra of the periodic structures with different CBP thicknesses in periodic samples: black curve (*d*_*CBP*_ = 10 nm), red curve (*d*_*CBP*_ = 15 nm), green curve (*d*_*CBP*_ = 20 nm) and blue curve (*d*_*CBP*_ = 30 nm). Dashed gray curve shows PL spectrum of the 50 nm bare CBP layer. (**b**) Ratios between lifetime of the CBP excited state in the bare CBP layer (*τ*_*CBP*_) and lifetime in the periodic structure (*τ*_*structure*_). Black squares show the ratios for the 10 nm CBP/15 nm Ag structure. Red circles show the ratios for the 15 nm CBP/15 nm Ag structure and olive triangles show the ratios for 20 nm CBP/15 nm Ag structure case. Blue square shows the ratio for the 30 nm CBP/15 nm Ag structure. Solid black curve demonstrates PL spectrum of the 15 nm CBP/15 nm Ag structure.

Room temperature photoluminescence spectra measured from the top of the aforementioned structure are presented in Fig. 7. The bare CBP layer shows a wide emission band in the frequency interval from 2.8 eV to 3.3 eV, and the emission spectrum almost does not depend on the emission angle. It can be seen that a periodic structure exhibits a trend of widening of the emission range compared to the bare emitting CBP while also growing distinct peaks which roughly correspond to plasmonic peaks in Fig. 4b. The positions of the basis peaks for different widths of CBP are nearly the same as in the bare CBP case (3.02 eV, 3.17 eV, 3.26 eV) but with a redistributed intensity according to the coupling with periodic structure.

**Figure 8:**
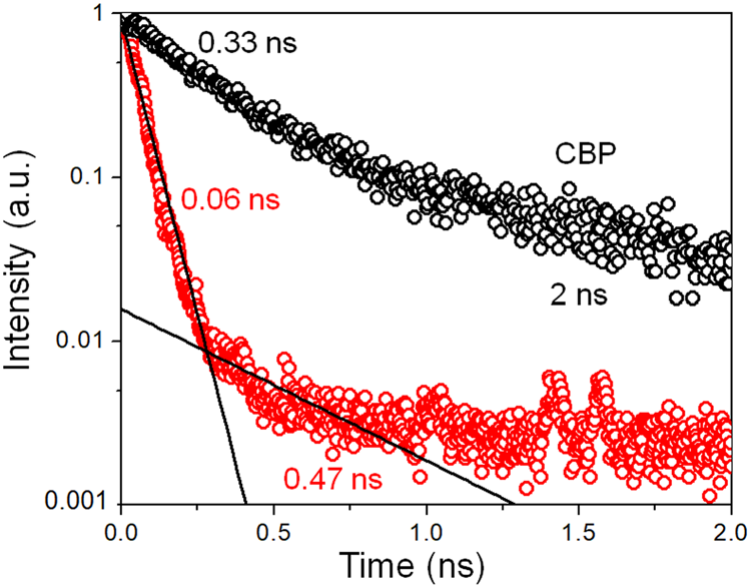
TCSPC measurements. PL decay time of bare CBP (black circles) and of periodic 20 nm CBP/15 nm Ag structure (red circles) taken at photon energy of 3.02 eV.

Further, we measured the lifetimes of excited states at certain energies. Fluorescence decay of CBP material is determined by two lifetimes and biexponential behavior as shown in Fig. 8 —an example of intensity decay curve for 20 nm CBP/15 nm Ag structure. The CBP molecule is characterized by “fast” (*τ*_*fast*_ ≈ 0.33 ns) and “slow” (*τ*_*slow*_ ≈ 2 ns) lifetimes and those parameters are constant within the error of measurement and approximation within the whole CBP emission range.

We define the experimentally measured Purcell factor as a ratio between the excited state lifetimes in bare CBP and in the periodic structure. In our case measurements showed that for every structure the ratios between “fast” lifetimes of a bare CBP and periodic structure are the same as the ratios between “slow” lifetimes within the range of the experimental error. Figure 7b demonstrates the distribution of lifetime ratios for various periodic structures at the energies corresponding to CBP emission range. Lifetime ratios’ magnitude is from 4 to 8; relatively high experimental error, especially at the edge of the CBP is due to low intensity of CBP emission at energies higher than 3.4 eV. This means moderate increase of spontaneous emission rate for the whole set of test structures. The interface between metal and CBP accounts for the majority of the decay, making it non-radiative. Taking this fact into account we can conclude that decrease in the excited state lifetime due to radiative decay in structures with various CBP thickness is still no less than 4.

## Conclusions

In summary, we have modelled, fabricated and studied periodic metal-dielectric structures. We have shown that contrary to previous propositions the giant values of the calculated Purcell factor are only achievable when absorption in metals is not considered. Using experimentally obtained refractive index spectra for silver we have calculated that in a silver/vacuum structure the values can barely exceed unity. However, by tuning away from high DOS region and using organic materials that emit light in the plasmonic band one can achieve a moderate increase in the Purcell factor that is about 10 times higher than in simple metal/vacuum model. We have backed this claim with experimental study of a periodic structure that had CBP as active region. We have found that the excited state lifetime is 4–8 times shorter in metallic structures compared to bare CBP.

## Methods

### Sample preparation

The set of periodic structure samples with different thickness of the CBP (10 nm, 15 nm, 20 nm and 30 nm) was fabricated using a Kurt J. Lesker® Spectros II™ system, an organic and metal thin film deposition system capable of reaching vacuum levels as low as 1 × 10^−6^ mbar. 5 periods of CBP and silver layers were deposited on the Al_2_O_3_ (sapphire) substrate. A set of calibration depositions for both materials was performed with greater accuracy. Also, a CBP layer of thickness 50 nm was grown on Al_2_O_3_ substrate for comparison of bare CBP properties with periodic structures properties.

### Optical measurements

Emission spectra of bare CBP layer and periodical structures were measured using a Jobin-Yvon Horiba®Fluorolog™ FL3-22 spectrometer (bare CBP and periodical structures were excited at 4.73 eV) at room temperature and atmospheric pressure.

Time-resolved measurements were performed using time-correlated single photon counting (TCSPC) measurement scheme at room temperature and atmospheric pressure. Structures was excited through the top silver layer at 262 nm wavelength, the third harmonic of the output from a Coherent® Mira™ 900F Ti:Sapphire oscillator tuned to 786 nm central wavelength, with a 76 MHz repetition rate. A 365 ± 10 nm bandpass filter with OD (optical density) >4 outside this region was used. Samples were excited at 45° angle to the substrate normal. Emission from samples was collected also at 45° angle to the substrate normal at various energies (from 3.02 eV to 3.4 eV which is inside the range of CBP molecule emission).

### Calculation techniques

To obtain the Purcell factor we start with deriving a formula for the emission probability. In an infinite structure with layers parallel to *xy* plane the coordinates in the *k*-vector space are (*k*_*x*_, *k*_*y*_, *K*), where *K* is the Bloch vector. The well-known equation for the emission probability is:

4 $$dW=\frac{2\pi }{\hslash }{|{V}_{fi}|}^{2}\delta ({E}_{i}-{E}_{f}-\hslash \omega )d\nu ,$$

where *dW* is the probability of a radiative transition between dipole states with energies *E*_*i*_ and *E*_*f*_. The transition is characterized by the matrix element *V*_*fi*_, the photon energy $\hslash \omega$ and the number of photon states *dν* that is proportional to a volume element in *k*-space and quantization volume *V* (which in an infinite structure spans one period):

5 $$d\nu =V\frac{d{k}_{x}d{k}_{y}dK}{{(2\pi )}^{3}}.$$

Since we are deriving the frequency Purcell factor we have to calculate ${\int }^{}dW$ for a constant *ω* and then divide the obtained emission probability by that of a free space (where *d* is the absolute value of the dipole moment):

6 $${W}_{0}=\frac{2{\omega }^{3}n}{3{c}^{3}\hslash }{|d|}^{2}.$$

This task is approached by replacing variables (*k*_*x*_, *k*_*y*_, *K*) with (*ω*, *k*_⊥_, *φ*) where *k*_⊥_ is the value of wavevector in *xy* plane and *φ* is the angle in this plane. This gives

7 $$d\nu =\frac{V}{{(2\pi )}^{3}}|\frac{dK}{d\omega }|{k}_{\perp }\,d\omega \,d{k}_{\perp }\,d\phi .$$

At this point it is important to consider the matrix element *V*_*fi*_:

8 $${V}_{fi}={\bf{e}}{\bf{d}}\langle f|E|\rangle i\approx d\,{\bf{e}}{\bf{r}}E({z}_{0})$$

Here we have assumed that the wavefunctions of initial and final states of the dipole overlap considerably only near the dipole position, *z*_0_. The values **e** and **r** are unit vectors of the electric field polarization and dipole orientation, respectively.

Both Equation 5 and Equation 7 reflect ambiguity in choosing the volume *V* because the number of states and the field amplitude depend on it; however, when the normalizing condition for the electric field amplitude

9 $$\frac{1}{4\pi }\int n{({\bf{r}})}^{2}{|E({\bf{r}})|}^{2}dV=\frac{\hslash \omega }{2}$$

is taken into account, we can part with *V* and postulate *V* ≡ 1 without it affecting the general derivation.

Substituting Equation 7 into Equation 4 and integrating over *ω* and *φ*, we get

10 $$d{W}_{\omega }({k}_{\perp })=\omega {|d|}^{2}\frac{{({\bf{e}}{\bf{r}})}^{2}{|E({z}_{0})|}^{2}}{2\pi \hslash }\,|\frac{dK}{d\omega }|{k}_{\perp }\,d{k}_{\perp }.$$

Finally, using Equation 6 we get the integral Purcell factor:

11 $$F(\omega )=\frac{\int d{W}_{\omega }({k}_{\perp })}{{W}_{0}}=\frac{3{c}^{3}}{4\pi {\omega }^{2}}\int {({\bf{e}}{\bf{r}})}^{2}{|E({z}_{0})|}^{2}|\frac{dK}{d\omega }|{k}_{\perp }\,d{k}_{\perp }.$$

In a finite structure such as a single interface, the procedure is very similar to the described above; however, we now deal with two dimensional *k* space, and after proper substitutions we obtain

12 $$d\nu =\frac{S}{{(2\pi )}^{2}}|\frac{d{k}_{\perp }}{d\omega }|{k}_{\perp }d\omega \,d\phi $$

Here *S* is quantization space (as opposed to quantization volume). Then, the normalization condition can be expressed as follow:

13 $$\frac{S}{4\pi }{\int }_{-\infty }^{\infty }n{(z)}^{2}{|E(z)|}^{2}dz=\frac{\hslash \omega }{2}$$

after that the ambiguity in choosing *S* is also resolved. Finally, the value of Purcell factor is

14 $$F(\omega )=\frac{3\pi {c}^{2}}{\omega \,}{k}_{\perp }(\omega )|\frac{d{k}_{\perp }}{d\omega }|{({\bf{e}}{\bf{r}})}^{2}{|E({z}_{0})|}^{2}.$$

## Data Availability

The experimental and modelling data are present in the paper. Additional data related to this paper may be requested from the authors.
